# How nursing informatization impacts nurses’ humanistic caring: a qualitative study based on Social Cognitive Theory

**DOI:** 10.3389/fmed.2026.1929882

**Published:** 2026-08-27

**Authors:** Yuan Liu, Yonglu Huang, Ao Huang, Xingyue Chen, Yuncai Chen, Li Li

**Affiliations:** 1 Department of Oral Nursing Teaching and Research, Nanjing Stomatological Hospital, Affiliated Hospital of Medical School, Nanjing University, Nanjing, China; 2 Institute of Stomatology, Nanjing University, Nanjing, China; 3 Medical School of Nanjing University, Nanjing, China; 4 School of Nursing, University of South China, Hengyang, China

**Keywords:** humanistic caring, nurses, nursing informatization, qualitative study, Social Cognitive Theory

## Abstract

**Objective:**

As nursing informatization continues to transform healthcare delivery, increasing attention has been directed toward nurses’ perceptions and experiences of the relationship between nursing informatization and humanistic caring. However, how nurses interpret and navigate humanistic caring within digitally transformed clinical environments remains insufficiently understood. This study aimed to explore nurses’ experiences and perceptions regarding nursing informatization and humanistic caring through the lens of Social Cognitive Theory (SCT).

**Methods:**

A qualitative study using SCT-guided directed content analysis was conducted. Semi-structured interviews were undertaken with 14 clinical nurses from a tertiary Grade-A hospital in Nanjing, China, between January and March 2026. Interview data were analyzed using SCT as an interpretive framework, focusing on nurses’ experiences across the personal, behavioral, and environmental dimensions.

**Results:**

Three themes and nine subthemes were identified: (1) subjective alienation and crisis (weakened motivation, changing perceptions, technology adaptation anxiety, and low self-efficacy); (2) behavioral reshaping and support (communication transformation, pattern differentiation, peer empowerment); (3) environmental double-edged sword (enhancing safety and care efficiency, systemic barriers and insufficient training).

**Conclusion:**

This study provides insights into nurses’ experiences and perceptions regarding nursing informatization and humanistic caring through the lens of Social Cognitive Theory. Rather than indicating a unidirectional effect, participants described nursing informatization as a contextual factor that may facilitate or constrain humanistic caring practices through complex interactions among cognitive, behavioral, and environmental factors. Humanistic caring in digital contexts appeared to involve ongoing adaptation and negotiation between technological demands and interpersonal values. These findings may inform future discussions and practice development on how healthcare organizations can support nurses in balancing technological competence with humanistic caring within digitally transformed environments.

## 1 Introduction

As global healthcare systems undergo profound digital transformation, the World Health Organization (WHO) emphasizes in its *Global Strategy on Digital Health* that technological innovation is a primary driver of achieving universal health coverage and enhancing healthcare efficiency (1). As the first year of *China’s The 15th Five-Year Plan*, 2026 marks a solid start for the implementation of health and wellness initiatives outlined in the plan. The 2026 National Conference on Health and Wellbeing in China emphasized the need to advance new quality productive forces in the health sector, strengthen innovation in health and medical technology, and promote digital and intelligent transformation (2). With the widespread adoption of electronic health records (EHRs) and artificial intelligence (AI) clinical decision support systems, the integration of nursing informatization has become an inevitable direction of development nursing practice (3). Intelligent nursing has demonstrated substantial social value in enhancing nursing efficiency, optimizing resource allocation, and improving patients’ quality of life, thereby contributing to the transformation of nursing care models (4–6). However, when efficiency-driven digital technologies are introduced into patient-centered nursing practice, nurses may experience data overload and face the risk of digital alienation (7, 8). In this context, technology may shift from being a supportive tool to an implicit mechanism of control, ultimately weakening the therapeutic relationship between nurses and patients (9). The pervasive use of digital technologies often generates considerable technostress among nurses, creating an invisible barrier between digital interfaces and patients’ lived experiences. Over time, this contributes to varying degrees of humanistic fatigue in highly digitized clinical environments (10). The humanistic care capacity of clinical nurses represents a core professional competency that cannot be fully replicated by machines or AI systems. The humanistic dimension of nursing further constitutes nurses’ professional identity and value. Particularly in the context of a global nursing shortage and an aging population, preventing the marginalization of humanistic care amid rapid technological advancement has become a critical challenge for nursing management worldwide (11). To address this issue, this study adopted Albert Bandura’s Social Cognitive Theory (SCT) (12), a fundamental framework in social psychology that has been widely applied in nursing research to explain behavioral change processes (13–15). Guided by this theory, the present study explored how clinical nurses perceive and interpret the relationship between nursing informatization and humanistic caring from personal, behavioral, and environmental perspectives.

## 2 Methods

### 2.1 Study design and participants

This study employed SCT-guided directed content analysis to explore clinical nurses’ perceptions regarding the impact of nursing informatization on their humanistic care capacity through semi-structured interviews. The aim was to explore clinical nurses’ experiences with nursing informatization use and to identify the factors underlying changes in their humanistic care capacity. A combination of purposive and snowball sampling methods was employed. Between January and March 2026, 14 clinical nurses from four departments—the Department of Thoracic and Cardiovascular Surgery, the Department of Geriatrics, the Outpatient Department, and the Nursing Department—at a tertiary Grade-A hospital in Nanjing, Jiangsu Province, were recruited to participate in the study.

#### 2.1.1 Inclusion and exclusion criteria

Inclusion criteria: (1) having at least 5 years of clinical work experience; (2) having received undergone standardized training and education on nursing informatization systems at the hospital; (3) possessing full capacity to communicate; (4) providing informed consent and voluntary participation in this study. Exclusion criteria: (1) trainee nurses or rotating nurses; (2) nurses unable to fully participate in the interview or express their views clearly due to physical, psychological, or work-related reasons.

#### 2.1.2 Determination of sample size

The sample size was determined based on the principle of information power (16), which suggests that the required number of participants depends not only on data saturation but also on the study’s aim, the specificity of the sample, the quality of dialog during interviews, and the depth of analysis. Sample saturation was determined based on the principle of data saturation, specifically when no new themes emerged from three consecutive interviews (17). Previous research by Guest et al. suggested that the required sample size for qualitative interviews could be achieved within approximately 12 interviews (18). A total of 18 nurses were invited to participate in this study. Among them, 14 agreed to participate and completed the qualitative interviews. Three nurses participated in pilot interviews; since these were primarily used to assess the validity, clarity, and feasibility of the interview guide (19) rather than to collect formal research data, their interview data were excluded from the final analysis. Starting with the 12th interview, no new themes emerged during three consecutive interviews. Therefore, 14 nurses were ultimately included. In addition, to maximize sample diversity, this study included clinical nurses from outpatient, internal medicine, and surgery departments to ensure diversity across personal (e.g., clinical knowledge and self-efficacy), behavioral (e.g., nurse–patient communication and work styles), and environmental (e.g., the level of departmental informatization), thereby enriching the diversity of experiences represented in the sample.

### 2.2 Data collection method

This study employed a qualitative design using SCT-guided directed content analysis of semi-structured in-depth interviews to explore clinical nurses’ perceptions and experiences regarding humanistic caring practices within the context of nursing informatization through semi-structured interviews. The semi-structured interview guide was developed based on the research objectives and theoretical framework, with the primary goal of eliciting rich and in-depth information from participants rather than simply increasing the number of questions (20). First, based on the research objectives and SCT framework, and following a comprehensive literature review, the interview guide was developed through research team discussions and expert guidance from the supervisor. Each core question was accompanied by follow-up probes designed to encourage participants to elaborate on their individual experiences. These follow-up questions were flexibly adjusted according to participants’ responses, rather than being applied in a rigid manner (21). This approach ensured that interviews remained focused on the research objectives while allowing novel and meaningful insights to emerge naturally. Second, three participants meeting the inclusion and exclusion criteria were selected for pilot testing of the interview guide through pilot interviews. Finally, based on the results of the pilot interviews, the research team held a meeting to refine the interview guide, resulting in the final interview version (Table 1).

**Table 1:** Semi-structured interview outlines.

Main interview questions	SCT dimension	Example probing questions
Please describe your understanding of nursing informatization?	Personal factors	What changes has nursing informatization brought to your daily clinical practice?
		What were your initial perceptions or feelings when you first encountered nursing informatization?
		Do you think nursing informatization has changed your understanding of nursing work? Could you describe this change in detail?
Please describe the advantages and challenges that nursing informatization brings to clinical practice?	Behavioral and environmental factors	In your opinion, does nursing informatization mainly facilitate nursing practice or create additional challenges? Why?
		Have you experienced anxiety, frustration, or helplessness when learning or applying new information technologies?
		Do you think nurses’ age, work experience, or technological competence affects their adaptation to nursing informatization? Why?
Please describe your understanding of humanistic care in nursing.	Personal factors	Which tasks in clinical work are carried out to implement the nursing humanistic care?
		What are the core humanistic care abilities in the field of nursing?
In your opinion, how will humanistic care in nursing change in the context of widespread nursing informatization?	Behavioral and environmental factors	Has nursing informatization influenced the way you communicate and interact with patients? Could you provide specific examples?
		Do you think information technology helps you identify patients’ needs more effectively or provide more individualized care? Why?
		How do you balance technological requirements with emotional support and humanistic caring in an informatized nursing environment?
In the context of nursing informatization, how do you think we can promote the development of nurses’ humanistic care skills?	Reciprocal determinism	What changes or improvements do nurses themselves need to make?
		What types of support should nursing managers or healthcare organizations provide?
		Do you think nursing education should incorporate more information technology-related training? Why?
		What are your expectations or suggestions regarding the integration of technological empowerment and humanistic nursing care in the future?

The interviews were conducted by two researchers (graduate students who had received qualitative research training and were proficient in interview techniques). Interviews were held in quiet settings such as demonstration classrooms and activity rooms within each department, ensuring privacy and minimizing external interruptions. The interviewer contacted participants in advance to schedule interviews, introduced the study purpose and significance, explained recording confidentiality and data protection procedures, and obtained written informed consent before the interviews commenced. During the interviews, the environment was maintained as quiet and comfortable to ensure participant comfort and privacy. One researcher conducted the interview and recorded field notes, focusing on participants’ tone, demeanor, facial expressions, and gestures. The other researcher maintained a neutral stance and assisted with follow-up questions and clarifications when necessary, while avoiding leading or suggestive language. Within 24 h after each interview, the audio recordings were transcribed verbatim and supplemented with corresponding field notes. The transcripts were returned promptly to participants for member checking. Each interview lasted approximately 40–60 min. After anonymization of all personally identifiable information, the transcripts were returned to participants for online member checking. Participants were invited to review the transcripts for completeness, semantic accuracy and potential transcription errors. They were also allowed to modify, supplement, or remove any content as necessary. After receiving participant feedback, the research team conducted a final verification of the transcripts before proceeding to data analysis. Data saturation was considered to have been reached when no new themes emerged during three consecutive interviews.

### 2.3 Research team

The research team was composed of experienced nursing experts and postgraduate nursing students, including one senior researcher, one intermediate-level researchers and four postgraduate students. The first and second authors were mainly responsible for the research design, interviews and data analysis. During the interviews, one researcher conducted the interview, while the other assisted with participant observation and field note-taking. The two interviewers were non-clinical administrative personnel and had no superior–subordinate relationship with the participating nurses. All researchers jointly participated in discussions to resolve disagreements. When consensus could not be reached, the final decision was made by the corresponding author (Li Li), an expert in nursing management and nursing education, to ensure the rigor and accuracy of the data analysis process. All team members had received training in qualitative research and possessed extensive clinical experience, ensuring the methodological rigor and reliability of the study.

### 2.4 Data analysis method

Qualitative directed content analysis was used for data analysis (22, 23). Data collection, organization, and analysis were carried out simultaneously. Within 24 h of each interview, the audio recordings were transcribed verbatim. Two master’s-level nursing students, both trained in qualitative research methods, independently reviewed the transcripts, identified meaningful content, and generated initial codes. Any unclear or ambiguous content was promptly clarified and confirmed with participants during the member-checking process. An in-depth interpretation and analysis of all collected data was conducted, including textual materials, nonverbal behaviors, and field notes. Guided by SCT, the study used personal, behavioral, and environmental factors as a theoretical coding framework to organize coded data and identify themes. Subsequently, relationships among themes were analyzed to establish the final thematic structure.

### 2.5 Rigor

This study followed the reporting guidelines for qualitative research (24). To ensure the trustworthiness of the study, strategies addressing credibility, dependability, confirmability, and transferability were implemented.

#### 2.5.1 Credibility

This study adopted semi-structured in-depth interviews to collect data, allowing participants to explore key issues in depth and ensuring that the findings reflected their authentic clinical experiences. Investigator triangulation and independent verification were implemented during transcription and thematic analysis. Two researchers with experience in qualitative research independently read the interview transcripts and completed initial coding. They subsequently compared their coding results and resolved discrepancies in codes, categories, and themes through discussion. When differences in interpretation arose between the researchers, they revisited the original interview data and consulted an expert in qualitative research until a consensus was reached. A third researcher with experience in qualitative research further reviewed the coding process and thematic structure to assess whether the research interpretations were adequately supported by the original data. In addition, reflective field notes were maintained throughout the study. After each interview, the two interviewers jointly prepared reflective notes documenting potential researcher bias, participants’ nonverbal cues, and contextual observations. Regular team discussions were conducted to refine subsequent interview strategies and enhance reflexivity.

#### 2.5.2 Dependability

The interview guide was refined through pilot interviews and revised according to participant feedback. Standardized data analysis procedures were followed. Guided by SCT, the research team repeatedly reviewed and compared the interview data to ensure analytical consistency.

#### 2.5.3 Confirmability

During the interviews, only neutral follow-up questions were asked, and no leading or suggestive comments were made. Throughout the analysis process, the researchers continuously traced each theme and subtheme back to the original interview data to ensure that every theme and subtheme could be supported by specific statements from the participants. In addition, all research materials, including anonymized interview recordings, transcripts, coding records, expert consultation records, and reflective notes, were securely archived for audit purposes.

#### 2.5.4 Transferability

The participants in this study were nurses from four different departments, including internal medicine and surgery, thereby enhancing the diversity of participant characteristics. In addition, representative participant quotations were provided for each theme and subtheme to illustrate the analytical findings and facilitate assessment of their transferability to similar clinical settings.

#### 2.5.5 Ethical considerations

This study was approved by the Ethics Committee of the stomatological Hospital, Medical School of Nanjing University (NJSH-2026NL-021). All participants were informed of the study purpose, interview procedures, data use, and privacy protection measures, and provided written informed consent prior to participation. Participants were informed that they could withdraw from the study at any time without providing a reason. No explanation was required for withdrawal. If participants withdrew, their data were immediately destroyed and their decision had no effect on their employment or clinical practice. All audio recordings and written records were used solely for research purposes and were not disclosed or used for any other purpose without participants’ permission.

## 3 Results

### 3.1 Demographic characteristics of the participants

A total of 14 eligible clinical nurses participated in this study (Table 2). All participants were female, ranging in age from 25 to 53 years, with an average age of 37.07 years. The years of work ranged from 5 to 33 years, with an average of 15.14 years. In terms of educational background, 11 participants (78.6%) held a bachelor’s degree, and 3 participants (21.4%) held a master’s degree. In terms of professional titles, there was 1 senior nurse (7.1%), 8 supervisor nurses (57.2%), 4 co-chief superintendent nurse (28.6%), and 1 chief superintendent nurse (7.1%).

**Table 2:** Basic information about the interviewees (*n* = 14).

No.	Age	Gender	Educational background	Years of work	Professional title	Department	Interview duration (min)
N1	37	Female	Bachelor’s degree	17	Supervisor nurse	Cardiothoracic surgery	57
N2	30	Female	Bachelor’s degree	8	Supervisor nurse	Cardiothoracic surgery	48
N3	25	Female	Bachelor’s degree	5	Senior nurse	Cardiothoracic surgery	54
N4	41	Female	Bachelor’s degree	20	Co-chief superintendent nurse	Cardiothoracic surgery	46
N5	35	Female	Bachelor’s degree	14	Supervisor nurse	Cardiothoracic surgery	41
N6	37	Female	Bachelor’s degree	15	Supervisor nurse	Geriatrics	53
N7	44	Female	Bachelor’s degree	21	Co-chief superintendent nurse	Geriatrics	57
N8	42	Female	Bachelor’s degree	20	Co-chief superintendent nurse	Geriatrics	42
N9	38	Female	Bachelor’s degree	16	Supervisor nurse	Geriatrics	46
N10	42	Female	Bachelor’s degree	20	Co-chief superintendent nurse	Outpatient department	42
N11	29	Female	Master’s degree	5	Supervisor nurse	Outpatient department	50
N12	31	Female	Master’s degree	7	Supervisor nurse	Outpatient department	55
N13	35	Female	Bachelor’s degree	11	Supervisor nurse	Outpatient department	41
N14	53	Female	Master’s degree	33	Chief superintendent nurse	Nursing department	45

### 3.2 Themes

Three main themes and nine subthemes were ultimately identified (Table 3): subjective alienation and crisis (weakened motivation, changing perceptions, technology adaptation anxiety, and low self-efficacy); behavioral reshaping and support (communication transformation, pattern differentiation, peer empowerment); environmental double-edged sword (enhancing safety and care efficiency, systemic barriers and insufficient training).

**Table 3:** Themes, subthemes, and concepts from the interviews.

Theme	Subthemes	Concepts
Subjective alienation and crisis	◆ Weakened motivation	I gradually depend on systems rather than my own judgment. (N2/N3) I complete tasks mechanically with reduced caring motivation. (N4/N10) I have less time and energy for humanistic care. (N1/N7)
	◆ Changing perceptions	I regard efficiency and precision as part of caring. (N12/N13) I feel care is becoming technology-centered. (N4) I believe humanistic care can coexist with technology. (N7/N14)
	◆ Technology adaptation anxiety	I feel stressed by rapid system updates. (N3/N11) I struggle to adapt under heavy workloads. (N6) I worry technology weakens my caring ability. (N1)
	● Low self-efficacy	I lack confidence in using complex systems. (N1/N4/N7/N14) I feel left behind by younger nurses. (N6/N7) I fear being replaced by technology. (N5/N8/N10)
Behavioral reshaping and support	◆ Communication transformation	I focus more on screens than patients. (N1/N11) Digital tools expand my caring methods. (N6/N8) Care is shifting toward data-driven interaction. (N10/N12)
	● Pattern differentiation	I sometimes know the data more than the patient. (N1/N4/N11/N14) Data analysis helps me provide proactive care. (N6/N7/N8) I balance technical logic with humanistic care. (N10/N14)
	◆ Peer empowerment	I learn caring behaviors from role models. (N1) Peer support helps me adapt to informatization. (N5) A positive atmosphere motivates my caring behaviors. (N8/N9/N12)
Environmental double-edged sword	● Enhancing safety and care efficiency	Informatization improves my efficiency and patient safety. (N3/N7/N6) Integrated data supports continuous care. (N5/N8/N10) I can spend more time communicating with patients. (N2/N12)
	● Systemic barriers and insufficient training	System problems increase my workload. (N1/N3/N7/N9) Training lacks practical value. (N3/N5/N8N14) I gradually become task- and system-oriented. (N5/N8)

“●” indicates that a subtheme was frequently mentioned, with 8 or more participants supporting it; “◆” indicates that a subtheme was moderately frequently mentioned, with 4–7 participants supporting it.

#### 3.2.1 Theme 1: subjective alienation and crisis (personal factors)

Nurses described that their experiences of humanistic care and professional perceptions were closely intertwined with their experiences with nursing informatization. Some nurses experienced changes in their motivation to provide care and changing perceptions, anxiety regarding adaptation to new technologies, and reduced self-efficacy. These phenomena reflect the complex tension between technological empowerment and humanistic values within nursing informatization, highlighting the tension between technological demands and humanistic values in nurses’ professional experiences.

##### 3.2.1.1 Weakened motivation

Some nurses described that increased reliance on information systems was accompanied by feelings of reduced professional autonomy and decreased motivation for proactive patient engagement. Highly developed information systems may also impair nurses’ critical thinking and clinical decision-making abilities, leading them to deliver care strictly according to system-generated templates and ultimately weakening their original caring intentions.

Representative quotations:

N2: “Some nurses believe that since machines can greatly improve work efficiency, they naturally become dependent on these systems. To be honest, I have had such thoughts as well.”

N10: “Neglect of patient care is indeed possible. In the past, we took blood pressure manually. Now, we may simply glance at the monitor, see that the blood pressure is normal, and walk away.”

N1: “I spend too much time on systems and devices every day. I get exhausted quickly and just want to finish my work (leaving insufficient time for humanistic care).”

N3: “I do exactly what the system tells me to do, there should not be any major problems.”

These quotes suggest that nurses’ reliance on information systems may influence their motivation to provide care, clinical reasoning processes, and initial caregiving intentions.

##### 3.2.1.2 Changing perceptions

Nurses’ understanding of humanistic care appeared to evolve alongside the implementation of nursing informatization. Under the influence of digitalized nursing practice, many participants increasingly conceptualized humanistic care in terms of precision, efficiency, and timely problem-solving. At the same time, some nurses perceived that contemporary humanistic care has gradually evolved toward technology-supported and human–machine collaborative care, reflecting the integration of technological support with interpersonal caring.

Representative quotations:

N12: “When patients need professional help, if we can use digital tools to resolve issues more quickly, is not that a better form of humanistic care?”

N13: “Our philosophy is professionalism with warmth, but professionalism comes first. The real need is to solve professional problems. As long as you can solve those problems, humanistic care is reflected invisibly.”

N4: “Comforting patients through physical touch is becoming increasingly rare.”

N7: “If the system handles risk identification for patients while I provide emotional support, does that not enrich care even further?”

These findings suggest that nursing informatization influences not only nurses’ clinical practice but also their perceptions of humanistic care, professional values, and the meaning they attribute to caring behaviors. Rather than viewing technology and humanistic care as mutually exclusive, many participants described an evolving understanding in which technological competence was regarded as an integral component of compassionate nursing practice.

##### 3.2.1.3 Technology adaptation anxiety

Frequent system upgrades and the simultaneous use of multiple digital platforms appeared to increase nurses’ learning burden and occupational stress. Participants described experiencing anxiety when adapting to rapidly evolving technologies, which may constrain their ability to express humanistic caring behaviors. In addition, individual differences in digital literacy, together with heavy clinical workloads, further influenced nurses’ ability to adapt to digital transformation.

Representative quotations:

N3: “The system updates too quickly, I cannot adapt.”

N11: “I’m completely at a loss!”

N1: “It’s not smart enough yet, many operations still have to be done manually.”

N6: “Frequent night shifts, piles of nursing records, only three people on duty in the department. I already have too much work. It’s too hard to adapt the system.”

These narratives suggest that participants’ concerns extended beyond difficulties in operating digital systems. Rather, they reflected broader psychological responses arising from rapid technological change, evolving professional roles, increasing workload, and the cognitive demands associated with continuous adaptation.

##### 3.2.1.4 Low self-efficacy

As nursing informatization continued to advance, younger and newly graduated nurses generally expressed greater confidence and willingness to learn emerging technologies. In contrast, more experienced nurses often reported lower confidence in adapting to digital systems, which may contribute to concerns about professional obsolescence and reduced professional value.

Representative quotations:

N14: “I used to be very experienced, but now, because of them (the constantly updated operating systems), I feel helpless—I’ve suddenly become a novice.”

N4: “They (the new nurses) learn so much faster than we older nurses do, I feel abandoned.”

N7: “If I could leave right now, I would. All the high-tech thing (systems and equipment) constantly makes me feel like I’m going to be replaced at any moment.”

N8: “When facing these complicated systems, I am always afraid of making mistakes.”

These findings indicate that participants’ reduced self-efficacy primarily stemmed from uncertainty about their ability to adapt to ongoing digital transformation and concerns regarding their continuing professional value. For many experienced nurses, the rapid pace of technological change challenged their confidence in maintaining competence within an increasingly digitalized clinical environment.

#### 3.2.2 Behavioral reshaping and support (behavioral factors)

This theme reflects changes in nurses’ caring behaviors, communication patterns, and professional practice following the implementation of nursing informatization. On the one hand, digital technologies have transformed nursing communication by extending care beyond traditional time and space boundaries. On the other hand, participants described concerns that increasing reliance on digital systems might reduce emotional engagement and individualized care. In addition, peer support emerged as an important resource that helped nurses adapt to nursing informatization while maintaining humanistic care practices.

##### 3.2.2.1 Communication transformation

With the widespread adoption of nursing informatization, participants described substantial changes in their daily clinical practice. Whereas nurses previously focused primarily on patients’ facial expressions and bedside interactions, they now spend more time interacting with digital interfaces. Increased documentation demands were perceived to reduce opportunities for direct emotional interaction with patients.

Representative quotations:

N10: “The chip-based monitoring system can directly capture vital signs at every moment and automatically upload them into our nursing records.”

N1: “It creates the illusion of ‘mindless’ operation, where we do not actually go to the patient’s bedside to observe their actual condition but merely complete the records mechanically.”

N11: “Before, I was face-to-face with patients, but now there is a computer screen between us.”

At the same time, participants also emphasized that digital technologies had expanded opportunities for caring by extending communication beyond conventional face-to-face encounters.

Representative quotations:

N6: “Bedside mobile screens allow patients to watch educational content presented in animated formats while lying in bed.”

N8: “The real-time medication inquiry function of the online hospital has made it much easier for us (outpatient nurses) to communicate with out-of-town patients regarding online registration, follow-up appointments, and medication collection.”

Participants described nursing informatization as changing not only the tools used in clinical practice but also the ways nurses communicate with patients. While digital technologies created new opportunities for health education and continuity of care, participants also expressed concern that increased dependence on digital systems might reduce direct interpersonal interaction during clinical care.

##### 3.2.2.2 Pattern differentiation

Participants described nursing documentation as an essential component of clinical practice and a foundation for individualized patient care. However, some nurses perceived that the convenience of informatized systems could encourage greater reliance on standardized documentation, occasionally reducing attention to individualized patient assessment.

Representative quotations:

N3: “Some records may simply be copied, causing discrepancies between certain patients’ actual situations and the records.”

N14: “I have known patients’ data, but I may not truly understand the patient.”

On the other hand, the widespread use of informatization has also enabled nurses to provide proactive care through data analysis, allowing them to deliver more targeted interventions throughout the care process.

Representative quotations:

N6: “We can pull up a patient’s data from several years ago and present it in a graph for easy visualization.”

N8: “After patients are discharged, we can provide long-term health guidance and remote psychological support.”

Furthermore, participants with different levels of clinical experience emphasized different aspects of nursing informatization. Less experienced nurses were concerned about the impact of information systems on traditional nursing work models, whereas experienced nurses are more concerned with how to maintain the humanistic care aspect of nursing in a technology-driven environment.

Representative quotations:

N11: “I think these technologies and systems mainly revolve around my daily work.”

N10: “Digitalization is creating a distance between me and my patients, this concern is more thought-provoking than the impact on routine work.”

Participants described both advantages and challenges associated with informatized nursing practice. While digital systems enhanced access to patient information, some nurses expressed concern that highly standardized workflows might reduce opportunities for individualized assessment and direct understanding of patients’ experiences. Similarly, when it comes to the application of information technology in nursing practice, nurses with varying levels of experience focus on different aspects.

##### 3.2.2.3 Peer empowerment

Many participants emphasized the importance of peer support and role modeling in adapting to nursing informatization. Observing colleagues’ caring behaviors and sharing experiences with digital systems were described as valuable sources of learning and professional support.

Representative quotations:

N1: “My colleagues have many strengths in communicating with patients that are worth learning from.”

N5: “We spontaneously organize regular exchange meetings to learn new systems together and resolve difficulties encountered during use.”

N8: “Last month, the hospital’s ‘Model Nurse for Humanistic Care’ spent her free time helping elderly patients sign in and pay fees at the self-service machine. These seemingly trivial acts truly reminded me that I should follow her example.”

N9: “The atmosphere in the department is so positive that it inspires me to share that warmth with my patients.”

Participants consistently described supportive colleagues and exemplary role models as important influences on both their caring behaviors and their adaptation to informatized practice. A positive team atmosphere was perceived to encourage ongoing engagement in humanistic care by strengthening nurses’ confidence and behavioral motivation.

#### 3.2.3 Environmental double-edged sword (environmental factors)

Participants described the nursing informatization environment as having both facilitating and constraining effects on clinical practice. While digital technologies were perceived to improve patient safety, work efficiency, and continuity of care, participants also reported that system failures, fragmented digital platforms, and insufficient training increased workload and hindered adaptation to digital practice.

##### 3.2.3.1 Enhancing safety and care efficiency

Participants generally perceived nursing informatization as improving clinical efficiency and enhancing patient safety. Digital technologies were described as facilitating access to comprehensive patient information, streamlining nursing workflows, and reducing routine repetitive tasks. Several participants also noted that the time saved through informatization created additional opportunities for meaningful communication with patients and strengthened continuity of care.

Representative quotations:

N7: “The smart mattress monitors patients’ vital signs, and I receive an alert when a patient attempts to get out of bed, which reduces the risk of adverse events.”

N10: “Digitalization ensures more comprehensive management and access to patient data, which is extremely helpful for follow-up visits and subsequent consultations.”

N3: “Electronic display screens outside wards, call bell systems, infusion systems, and these technologies have greatly improved the clinical care environment and facilitated my nursing work.”

N2: “Once I became proficient with the system, I could spend more time listening to patients’ concerns.”

N5: “Through the follow-up system, I have remained in contact with a discharged patient for five years now. He is doing well in his daily life, and this gives me a strong sense of accomplishment.”

Participants consistently described nursing informatization as enhancing patient safety, improving work efficiency, and supporting continuity of care. Many also perceived that reduced administrative burden created more opportunities for patient communication and sustained caring relationships.

##### 3.2.3.2 Systemic barriers and insufficient training

Participants reported that technical problems within hospital information systems and insufficient integration across digital platforms created additional challenges in their daily practice. When information systems malfunctioned or were poorly interconnected, nurses often needed to undertake additional work, reducing the time and energy available for humanistic caring practices. Participants also reported that training provided by hospitals and departments during the implementation and optimization of digital systems often lacked depth and practical relevance.

Representative quotations:

N7: “When system failures occur, we rely on vendors for timely maintenance. Our internal capacity to resolve these problems is limited, and this seriously affects our work.”

N9: “We have to document every nursing procedure and action. This information is scattered across different systems, and the doctors’ and nurses’ workstations aren’t synchronized, so updates aren’t real-time. This can easily lead to problems with patient records.”

N3: “Hospital training always focuses on vague theoretical knowledge. There’s very little content that actually helps me do my job.”

N14: “These trainings are merely superficial initiatives and do not truly incorporate the humanistic experiences of clinical nurses, not even a little bit.”

Participants consistently identified fragmented information systems, technical failures, and insufficient training as environmental barriers to nursing informatization. These challenges were perceived to increase workload and learning demands while reducing the time and opportunities available for delivering humanistic care.

## 4 Discussion

**Figure 1:**
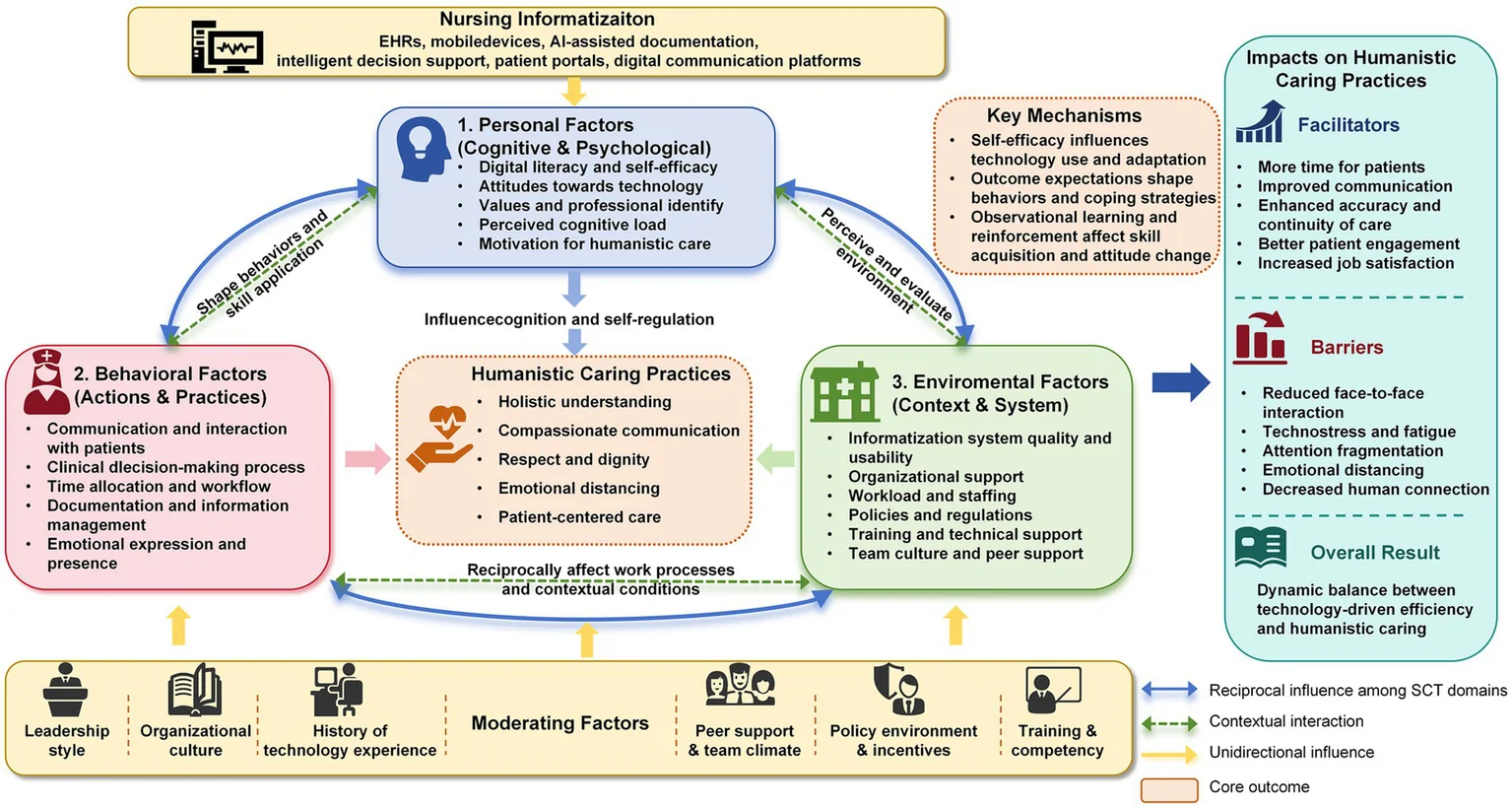
Social cognitive theory-guided model of how nursing informatization influences nurses’ humanistic caring practices: A triadic reciprocal determinism framework. EHRs, electronic health records; AI, artificial intelligence; SCT, social cognitive theory.

The findings suggest that nurses’ experiences of humanistic care in informatized healthcare settings involve interconnected personal perceptions, caring behaviors, and environmental contexts. Rather than representing isolated changes in caring behaviors, participants described a dynamic process of adaptation involving cognitive perceptions, behavioral responses, and environmental influences. These findings can be interpreted through the lens of SCT, particularly the principle of triadic reciprocal determinism, which provides a useful framework for understanding the reciprocal interactions among nurses’ perceptions, caring behaviors, and clinical environments in digitally transformed healthcare contexts (Figure 1).

At the personal level, participants described challenges related to self-efficacy when adapting to digitally transformed clinical environments. These experiences were not simply related to perceived technical competence but were also associated with nurses’ perceptions that digital systems were becoming increasingly involved in clinical decision-making, requiring nurses to balance technology-supported procedures with professional judgment. Participants also described technology adaptation anxiety and concerns about professional displacement, which they perceived as potential challenges to maintaining humanistic caring practices. These findings are consistent with previous research by Zhou et al. (25). Frequent system updates, increasing information overload, and complex digital workflows may further contribute to cognitive burden, thereby reducing nurses’ sense of control over their professional competence. Previous studies have shown that the use of electronic health records (EHRs) is significantly associated with psychological stress among nurses, particularly when system complexity is high and usability is limited (26). This finding is also consistent with the emerging concept of digital alienation, which suggests that highly technologized healthcare environments may alter perceptions of professional autonomy, identity, and emotional connection in clinical practice (27).

From the perspective of SCT, self-efficacy is considered an important personal factor that may influence individuals’ engagement in behaviors and adaptation processes. Therefore, supporting nurses’ confidence in using digital technologies may be an important consideration for facilitating adaptation to digitally transformed clinical environments while maintaining humanistic caring values. As Zhao argued, technological advancement should be viewed as a supportive tool rather than a replacement for the humanistic dimensions of nursing (28). Importantly, nurses should be encouraged not only to use digital technologies but also to become active participants in their implementation and optimization. In the present study, some participants reported concerns regarding excessive reliance on information systems, which they perceived as potentially reducing their attention to patients’ individualized needs. This finding is consistent with Kim et al. (29), who suggested that introducing new technologies without adequate critical thinking may compromise nurses’ clinical judgment. Consequently, healthcare organizations should strengthen digital literacy education, technology-supported decision-making training, and critical thinking development. Enhancing nurses’ self-efficacy and critical thinking capabilities may represent important areas for supporting adaptation to digital technologies while preserving attention to individualized, patient-centered humanistic care practices.

At the behavioral level, nurses’ caring behaviors may interact reciprocally with cognition, self-efficacy, and outcome expectations. In this study, participants’ accounts illustrated how changes in informatized clinical practices were associated with their perceptions and experiences of humanistic caring. The present study suggests that nurses perceived nursing informatization as being associated with changes in caring-related practices, involving both facilitating and constraining experiences. Participants described a gradual transition from traditional face-to-face nurse–patient communication toward platform-based information exchange, which could reduce opportunities for direct emotional engagement. Previous studies have similarly reported that the widespread use of electronic health records (EHRs) increases nurses’ documentation burden and reduces time available for direct patient interaction (30). From the perspective of SCT, such negative behavioral feedback may influence reliance on system-generated information while diminishing the perceived importance of bedside observation and interpersonal communication.

Conversely, other participants emphasized that nursing informatization was perceived as providing new opportunities for implementing caring behaviors. Information systems facilitate multimedia health education, remote communication, follow-up support, and continuity of post-discharge care delivery. Similar findings have been reported by Johnson et al. (31). Participants’ positive experiences with digital tools appeared to support their confidence in incorporating digital approaches into caring practices. Rather than viewing informatization solely as a challenge, some nurses described it as a potential resource for expanding opportunities for communication, follow-up, and patient support. Furthermore, the findings suggest that nurses’ experiences of humanistic care in digital healthcare environments may involve not only individual practices but also interactions with colleagues and organizational environments. Consistent with this observation, a phenomenological study reported that effective teamwork is fundamental to communication with family caregivers during end-of-life care (32). Within the framework of SCT, nurses’ caring behaviors can be understood as being shaped through interactions among personal factors, observational learning, social influences, and organizational contexts. Likewise, previous research has shown that peer learning strategies play a critical role in facilitating nurses’ professional development and adaptation to changing clinical practices (33).

From a practical perspective, the findings highlight the potential importance of supportive feedback processes within nursing informatization for helping nurses maintain attention to humanistic care. Healthcare organizations may consider developing more comprehensive feedback approaches that incorporate nurses’ experiences, patient perspectives, and communication-related aspects alongside existing nursing performance indicators. Peer support and role-modeling opportunities may also represent valuable approaches for facilitating nurses’ learning and adaptation in digital caring practices. Nurses with greater confidence and experience in integrating digital tools into caring practices may serve as valuable resources for peer learning by sharing approaches to digital communication, health education, and continuity-related care activities (34, 35). Finally, incorporating patient feedback into digital nursing systems may provide additional perspectives for nurses and organizations to reflect on caring practices and identify areas for improvement within digitally enabled healthcare environments.

At the environmental level, SCT provides a framework for understanding how environmental conditions may interact with individual cognition and self-efficacy in shaping behavioral experiences. In the present study, participants described how environmental conditions within informatized clinical settings were closely related to their experiences of providing humanistic care. On the one hand, participants perceived that information systems could support aspects of patient safety, workflow organization, and continuity-related care. Some nurses described that digital tools helped them access information more efficiently and created possibilities for more patient-centered interactions. Similar findings have been reported in previous studies (36). On the other hand, participants consistently identified system failures, fragmented digital platforms, and insufficient training as persistent environmental challenges that they perceived as contributing to additional workload, psychological burden, and barriers to caring practice (37). Previous research has reported associations between mobile electronic health record use, perceived time pressure, and system-related stress among nurses, particularly in complex digital environments (38). Other studies have also described challenges related to modifying and retrieving electronic nursing documentation after initial data entry (39).

From the perspective of SCT, these findings highlight the potential value of supportive practice environments in understanding the reciprocal relationships among environmental conditions, individual cognition, and caring behaviors. Healthcare organizations may consider several complementary strategies to address these environmental challenges. First, timely technical support mechanisms should be established to minimize workflow disruptions associated with system failures and interoperability problems. Second, scenario-based digital nursing training should replace one-time operational instruction with continuous competency development, thereby strengthening nurses’ technological self-efficacy and behavioral adaptability. In addition, further developing informatics specialist nurses may enhance organizational informatics capacity while supporting colleagues’ digital competence (40). Finally, psychological support should be integrated into informatized nursing environments through routine psychological support services, digital stress monitoring, and organizational support strategies that help alleviate technology-related occupational stress. Collectively, these considerations may contribute to a more supportive practice environment and provide conditions for nurses to negotiate the relationship between digital technologies and humanistic caring practices.

Beyond the explanatory framework of SCT, the findings may also be explored through a philosophical lens to further understand nurses’ experiences of practicing humanistic care within digitally transformed healthcare environments. Participants’ experiences suggest a perceived tension between technological rationality and humanistic rationality, particularly in relation to the increasing presence of standardized digital workflows and nurses’ concerns regarding opportunities for interpersonal connection and emotional engagement. From the perspective of technological rationality, participants’ accounts reflected how digital nursing practices increasingly emphasize efficiency, standardization, and digital workflow management. Participants recognized that digital practices may provide opportunities for efficiency and standardization, while also raising concerns about maintaining the emotional, embodied, and relational dimensions of humanistic nursing. Importantly, the findings do not suggest that humanistic care is being replaced or diminished entirely. Instead, participants’ accounts indicate that caring practices are being interpreted and adapted within technologically mediated clinical environments, as nurses seek to balance technological efficiency with humanistic values.

These findings have important implications for nursing education and professional development. Educational systems should consider curriculum reform that integrates medical humanities, philosophy of technology, digital ethics, and nursing informatics. As emphasized by Flaubert et al. in the National Academy of Sciences report, nursing education for the digital era should integrate technological competence with ethical reasoning and humanistic values to address the changing nature of clinical relationships (41). Scenario-based simulation, digital ethics case discussions, and reflective learning activities may provide opportunities for nurses to critically examine the tension between technological and humanistic rationality while strengthening their ability to deliver compassionate care in digitally enabled clinical settings.

Nevertheless, participants’ experiences suggest that educational initiatives alone may not fully capture the broader organizational and contextual factors influencing how nurses navigate technological demands and humanistic values within healthcare environments. The OECD reported that most national digital health evaluation frameworks continue to prioritize efficiency, service coverage, and data quality while giving relatively limited attention to patient experience and the quality of caring relationships (42). Such evaluation systems may contribute to a greater emphasis on technological performance than relational aspects of care within healthcare organizations. Future digital health evaluation frameworks should therefore incorporate indicators of patient experience, communication quality, and humanistic care alongside conventional measures of efficiency and technological performance (43). This direction is consistent with recent digital health strategies adopted across Europe and OECD countries, which emphasize that digital transformation should strengthen both workforce capacity and supportive work environments rather than focusing solely on technological implementation (44). Collectively, these perspectives highlight the value of a human-centered approach to digital healthcare governance, in which technological innovation and humanistic care are considered interconnected dimensions rather than mutually exclusive priorities.

## 5 Limitations

This research is a qualitative one and has some limitations that need to be improved in future studies. First, the sample in this study was drawn from a single tertiary hospital. Although standardized inclusion and exclusion criteria were used to screen study subjects, differences among healthcare institutions in various regions—including variations in the allocation of medical resources, clinical management processes, and nursing models—may limit the generalizability and applicability of the study results to other settings. Therefore, it is necessary to conduct future multicenter studies that include clinical nurses from different regions and institutions at various levels of care to further validate the findings of this study and enhance the generalizability of the results. Secondly, this study is a qualitative one. Data was collected through semi-structured interviews, and subjective factors may have influenced the process of data collection and processing. Thirdly, there may be an interviewer effect. Researchers’ own prior cognitive tendencies toward informatization may unintentionally generate subtle suggestive expressions during communication. Fourth, the research subjects of this study are from China. The interviews were conducted in Mandarin, while the data analysis and article writing were carried out in English, which may lead to bias. Finally, the perspective of this study focused on nurses, and the sample size was small, not including patients, doctors, and hospital administrators, which might lead to incomplete research results. Therefore, in the future, the sample size in different regions should be expanded to conduct multi-perspective views on the application of nursing informatization for patients, doctors, hospital administrators.

## 6 Conclusion

This study explored nurses’ experiences and perceptions regarding nursing informatization and humanistic caring practices through the lens of Social Cognitive Theory. The findings suggest that nurses’ experiences of humanistic caring in informatized environments involve complex interactions among personal perceptions, caring behaviors, and organizational contexts. Nursing informatization was perceived as a contextual factor that may provide new opportunities for care delivery while also presenting challenges to maintaining interpersonal connections. These findings contribute to a deeper understanding of how nurses negotiate humanistic caring practices within digitally transformed healthcare environments and may inform future efforts to develop nursing informatics strategies that balance technological advancement with human-centered care values.

## Data Availability

The original contributions presented in the study are included in the article/supplementary material, further inquiries can be directed to the corresponding author/s.
